# Feather mite abundance varies but symbiotic nature of mite‐host relationship does not differ between two ecologically dissimilar warblers

**DOI:** 10.1002/ece3.3738

**Published:** 2017-12-20

**Authors:** Alix E. Matthews, Jeffery L. Larkin, Douglas W. Raybuck, Morgan C. Slevin, Scott H. Stoleson, Than J. Boves

**Affiliations:** ^1^ Department of Biological Sciences Arkansas State University Jonesboro AR USA; ^2^ Department of Biology The University of Texas at Tyler Tyler TX USA; ^3^ Department of Biology Indiana University of Pennsylvania Indiana PA USA; ^4^ Department of Forestry, Wildlife, and Fisheries University of Tennessee Knoxville TN USA; ^5^ United States Department of Agriculture Forest Service Northern Research Station Forestry Sciences Laboratory Irvine PA USA

**Keywords:** feather mites, host‐symbiont interactions, Parulidae, Proctophyllodidae, symbiosis

## Abstract

Feather mites are obligatory ectosymbionts of birds that primarily feed on the oily secretions from the uropygial gland. Feather mite abundance varies within and among host species and has various effects on host condition and fitness, but there is little consensus on factors that drive variation of this symbiotic system. We tested hypotheses regarding how within‐species and among‐species traits explain variation in both (1) mite abundance and (2) relationships between mite abundance and host body condition and components of host fitness (reproductive performance and apparent annual survival). We focused on two closely related (Parulidae), but ecologically distinct, species: *Setophaga cerulea* (Cerulean Warbler), a canopy dwelling open‐cup nester, and *Protonotaria citrea* (Prothonotary Warbler), an understory dwelling, cavity nester. We predicted that feather mites would be more abundant on and have a more parasitic relationship with *P. citrea*, and within *P. citrea*, females and older individuals would harbor greater mite abundances. We captured, took body measurements, quantified feather mite abundance on individuals’ primaries and rectrices, and monitored individuals and their nests to estimate fitness. Feather mite abundance differed by species, but in the opposite direction of our prediction. There was no relationship between mite abundance and any measure of body condition or fitness for either species or sex (also contrary to our predictions). Our results suggest that species biology and ecological context may influence mite abundance on hosts. However, this pattern does not extend to differential effects of mites on measures of host body condition or fitness.

## 1 INTRODUCTION

Many organisms engage in intimate relationships (symbioses) with other species, and these symbiotic relationships are commonly categorized as parasitic, commensal, or mutualistic. However, despite simple static categorization, these relationships may actually vary among closely related species and may be temporally or spatially dynamic (Chamberlain, Bronstein, & Rudgers, 2014; Thompson & Cunningham, 2002). Symbioses can occur on a transitional continuum, and there may be plasticity of the static symbiotic categorizations depending on context (Leung & Poulin, 2008). Birds harbor a variety of ectosymbionts including feather mites (Astigmata: Analgoidea, Pterolichoidea), whose symbiotic relationship with their avian hosts has recently been debated (Galván et al., 2008, 2012; Soler et al., 2012). Feather mites are obligatory ectosymbiotic arthropods that inhabit the small spaces between feather barbs and are thought to primarily feed on oily secretions from the uropygial gland, which are distributed across feathers by preening (Proctor, 2003). Feather mites have streamlined bodies and specialized ambulacra (feet) that allow them to hold tightly onto feather barbules and resist turbulent airflow during flight (Dabert & Mironov, 1999).

The specialized dietary and morphological adaptations of feather mites suggest the strong symbiotic relationship between mites and their avian hosts. However, the specific nature of this relationship (positive, negative, or neutral) and any context dependency of the symbiosis has not been resolved. Thus, few generalizations can be made about factors that drive variation in this relationship both within and among species.

Most previous work exploring this relationship has tested for correlations between mite abundance and current physiological condition. For example, correlations between feather mite abundance and host body mass and other body condition indices have led to inferences of both mutualism (Blanco & Frías, 2001; Lindström et al., 2009; Villa, Le Bohec, Koop, Proctor, & Clayton, 2013) and commensalism (Blanco, Tella, & Potti, 1997; Carleton & Proctor, 2010; Davis & Cornelius, 2013). Furthermore, previous studies typically have concentrated on how mite abundance can influence a single component of host current fitness (Dowling, Richardson, & Komdeur, 2001; Galván & Sanz, 2006; Galván et al., 2012), limiting the conclusions that can be drawn about the nature of the symbiosis. This is also limiting because a lag effect of mites is possible, and few studies have taken the next step to relate mite abundance to future host fitness (reproductive performance and/or annual survival), which may demonstrate how mites affect an individual over its lifetime. In fact, to our knowledge, only one study (Pap, Tökölyi, & Szép, 2005) has assessed how feather mite abundance relates both to reproductive parameters and annual survival; they found no relationships in either case.

These individual cases also highlight a major void in our understanding of relationships between feather mites and their hosts: Although a number of studies have explored the potential factors that are related to variation in abundance of feather mites on hosts within a species, few studies have then assessed how those same factors contribute to context dependency of the effects of feather mites on host fitness among and within species. A variety of among‐ (e.g., ecological affiliations) and within‐species factors (e.g., age or sex) may be related to mite abundance, and some may then interact with mite abundance to influence host fitness. Among species, some hosts may have greater mite abundances because of their ecological context or life history strategy (Diaz‐Real et al., 2014; Galván et al., 2008), which in turn can influence how mites can affect individual host fitness. This may be especially true if there is a threshold at which hosting mites becomes burdensome (Galván et al., 2008; Haribal, Dhondt, Rosane, & Rodriguez, 2005). Alternatively, individuals of some species may be able to sustain an equivalent mite abundance with no effects on fitness.

For example, a species’ nesting ecology may influence both mite abundance and the effects of mites on host fitness, as feather mites are dependent upon the microclimate of the host (and ultimately, the host's environment). During the breeding season, much of the host's environment is at the nest, especially for females. In addition, it is at the nest where feather mites primarily disperse to new hosts (the offspring; Doña et al., 2017), which means that they would be even more affected by the nest environment as they move from host to host. Specifically, understory dwelling, cavity‐nesting species may occupy nests that make them more susceptible to parasitic mite abundances than their canopy dwelling, open‐cup nesting counterparts (Galván & Sanz, 2006). This is mechanistically possible for several reasons. First, abiotic conditions, such as temperature and humidity within cavities in the understory, may be more suitable for mites, and thus hosts may have greater mite abundances (at least during the breeding season). This is because the greatest abundance of mites on individuals occurs at relatively high temperatures (above 20°C; Wiles et al., 2000) and many ectosymbionts increase in abundance as relative humidity increases (Moyer, Drown, & Clayton, 2002). Furthermore, secondary cavity‐nesting species (those that nest in cavities that have been made by heterospecifics) may be even more susceptible to parasitic mite abundances because of the potential for mites to live in previously used cavities and transfer to new hosts, a possibility that has been proposed but not yet tested (Carleton & Proctor, 2010). Finally, species with this life history strategy (secondary cavity‐nesting) could also increase the probability of a foreign mite species being horizontally transmitted to an evolutionarily naïve host, and the resulting incipient species‐interaction may fall further on the parasitic side of the symbiotic continuum (Johnson, Graham, & Smith, 1997; Leung & Poulin, 2008).

Just as interspecific variation in ecology may be related to both the abundance of feather mites and their effects on host fitness, intraspecific traits (e.g., sex and age) may also be important. Although variation in mite abundance by host sex has been investigated before (Carleton & Proctor, 2010; Hamstra & Badyaev, 2009), no obvious patterns have emerged and there is currently no clear explanation as to why this variation may exist. Here again, a species’ ecological context may play a role. For example, in species that exhibit typical sex roles, females may harbor greater mite abundances than their male partners because they spend more time on the nest laying and incubating eggs and brooding their young. They may also be more negatively affected by mites, especially if they harbor mite abundances above some threshold during the breeding season. Age may also be a factor, but there are conflicting patterns in the literature. For example, in Barn Swallows (*Hirundo rustica*), adults had higher feather mite abundances than juveniles (Pap et al., 2005). This result can be justified because mites typically mature one egg at a time (Dubinin, 1951), and it may take time for mite populations to build up on young birds. However, Davis and Cornelius (2013) found the opposite pattern, with younger House Finches (*Haemorhous mexicanus*) harboring more mites than adults. In addition, it is unknown how the interaction between ecological affiliation, age, and sex may influence feather mite abundance or feather mite impacts on host fitness.

In this study, we explored how feather mite abundance varied among species (due in part to differing nesting ecologies) and within species (by age and sex), and subsequently how these among‐ and within‐species factors mediated the nature of the symbiotic relationship between feather mites and their avian hosts (i.e., if mites have differential effects on hosts). To do so, we quantified feather mite abundance and corresponding fitness (reproduction and survival) from individuals belonging to two relatively closely related New World warbler species (family Parulidae) that differ in nesting ecology (one is a canopy dwelling, open‐cup nester and one an understory dwelling, secondary cavity nester). We tested two main hypotheses related to (1) the factors that explain variation in mite abundance among and within species, and (2) the relationship between mite abundance and host body condition and fitness components. We first hypothesized that mite abundance differs among and within species. We predicted that mites will be more abundant on (a) an understory dwelling cavity‐nesting species; and within species, on (b) females, and (c) older birds. Second, we hypothesized that the relationship between mite abundance and body condition and host fitness (reproductive performance and survival) is also contingent on several of these among‐ and within‐species factors. We predicted that relationships between mite abundance and (a) body condition and (b) host fitness will be more strongly negative (i.e., mites will have a more parasitic effect) for understory dwelling, cavity‐nesting species than for canopy dwelling, open‐cup nesters, and even more so for female cavity nesters than for conspecific males. We evaluated both reproduction and annual survival of individuals and we quantified feather mite abundance using a novel, objective system that included all primary and rectrix feathers.

## 2 MATERIALS AND METHODS

### 2.1 Avian study species

We focused our efforts on two relatively closely related songbirds in the family Parulidae (Lovette et al., 2010): *Protonotaria citrea* (Prothonotary Warbler) and *Setophaga cerulea* (Cerulean Warbler). The life histories of these species overlap in many respects: Both species are highly insectivorous, sexually dimorphic, socially monogamous, and nest in forests of the eastern United States (Buehler, Hamel, & Boves, 2013; Petit, 1999). Moreover, these species are both Neotropical–Nearctic migrants. However, these species differ in two important ecological factors: *P. citrea* is one of two warbler species that nest in cavities in the understory, <4 m above the ground (Petit, 1999); *S. cerulea* build open‐cup nests high in forest canopies, typically >15 m above the ground (Buehler et al., 2013). The molt schedule for the feathers that we assessed (primaries and rectrices) is nearly identical for both species; they both typically molt these feathers postbreeding, but before fall migration (or occasionally during early stages of migration in North America; Pyle, 1999; Boves, Fairhurst, Rushing, & Buehler, 2016; Erik Johnson, Audubon Louisiana, personal communication).

### 2.2 Study areas

**Figure 1:**
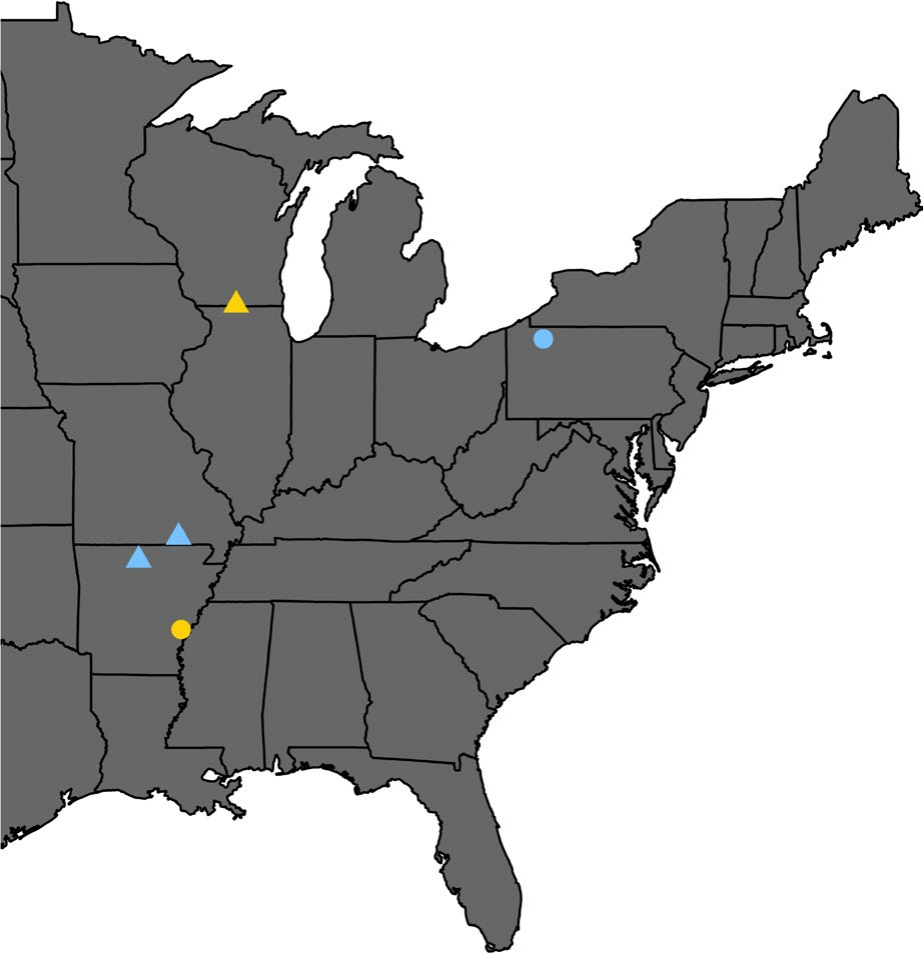
Map of primary (circles) and secondary (triangles) study sites in the eastern United States. The colors represent each host species (yellow: *Protonotaria citrea*; blue: *Setophaga cerulea*). This map was created with the R package “ggmap” (Kahle & Wickham, 2013)

We conducted our research during the breeding seasons of 2015–2016 at primary field sites that were located in areas where we had already been conducting unrelated research on these two warbler species. We then augmented these locations with secondary field sites during the following breeding season (in 2017). For *P. citrea*, our primary field site was in a southern portion of their breeding range in 100‐ha of east‐central Arkansas, USA in the Dale Bumpers White River National Wildlife Refuge (34°2′N, 91°1′W; Figure 1), where males and females both arrive by late April. For *S. cerulea*, our primary field site was in the northern portion of their breeding range in 500‐ha of northwestern Pennsylvania, USA, along the Allegheny River, extending onto the Allegheny Plateau (41°7′N, 79°2′W; Figure 1), where males and females both arrive by late May. These primary locations from which we collected data are clearly geographically separated, but these species only spend two to five months of their full annual cycle in these locations (Buehler et al., 2013; Petit, 1999). Evidence from data obtained by light‐level geolocation suggests that during the rest of the year (nonbreeding), many individuals from these two populations spend six to nine months relatively close to one another in northern Colombia (Tonra et al., in review; T.J.B. and D.W.R., unpublished data). Conversely, *S. cerulea* that breed closer to Arkansas appear to overwinter much further southwest along the Andes mountains (within or near Peru; D.W.R., unpublished data). Thus, when considering the full annual cycle, the breeding populations utilized for this study likely represent greater geographic similarity for a much longer time period than had we used individuals whose breeding locations were closer. Despite this likely overlap of nonbreeding locations, we further addressed the potential confounding factor of geography by adding secondary field sites for both species (in 2017). At these locations, we collected data on feather mite abundance, but due to logistical constraints, were unable to include reproductive or annual survival data in these areas. For *S. cerulea*, our secondary field site was in the southern portion of their breeding range in north‐central Arkansas, USA in Buffalo River National Park (36°0′N, 92°6′W; Figure 1) and in southeastern Missouri, USA along the Eleven Point River in Mark Twain National Forest (36°7′N, 91°2′W; Figure 1). For *P. citrea,* our secondary field site was in the northern portion of their breeding range in south‐central Wisconsin, USA in Avon Bottoms State Natural Area (42°5′N, 89°3′W; Figure 1).

### 2.3 Capturing birds

We captured birds at both primary and secondary field sites. To capture males of both species, we placed speakers and a decoy in vegetation on both sides of a mist net, and then broadcasted audio tracks of each species’ song or call. To capture female *P. citrea*, we held a mesh bag over nest cavity openings early in the incubation period and flushed the female into the bag. Once captured, we banded individuals with United States Geological Survey aluminum bands and a unique combination of plastic color bands (to allow for identification of individuals without recapture). Recorded data included sex (via plumage and brood patch/cloacal protuberance), age (via plumage or molt limits; SY: second year; ASY: after second year; Pyle, 1999), mass (using a digital scale), and wing chord (using a wing rule). All individuals were captured either just before or during the nesting period. Banding and animal handling procedures were permitted and approved by the USGS Bird Banding Lab Permit #23877 and Arkansas State University IACUC Protocol #638636.

### 2.4 Feather mite identification

To document feather mite identities, we collected a small number of mites from the primary and rectrix feathers of both warbler species (from individuals not included in this study). We sorted mite morphospecies using a dissecting microscope, and slide‐mounted representative specimens that we examined using a compound microscope. We used Gaud and Atyeo (1996) to identify specimens to genus, and Drs. Sergey V. Mironov (Zoological Institute, Russian Academy of Sciences) and Heather C. Proctor (University of Alberta) confirmed identification.

### 2.5 Measuring mite abundance

**Figure 2:**
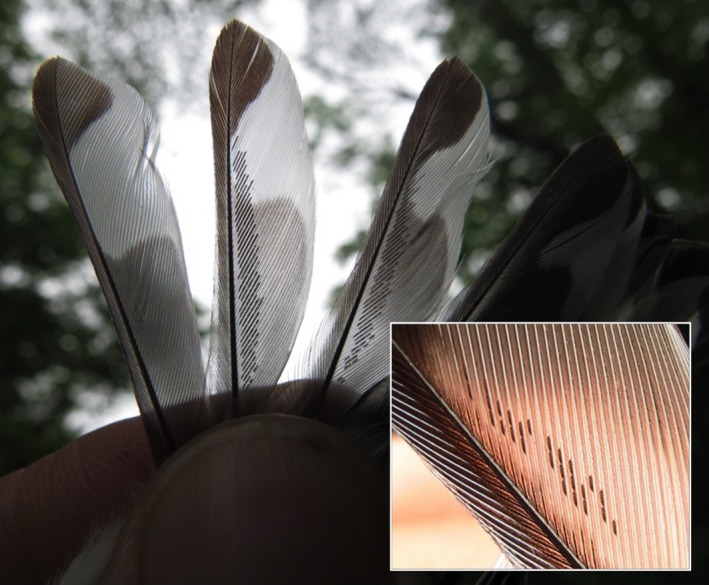
Procedure for objectively quantifying feather mite abundance on feathers. The feathers (either primaries or rectrices) are outstretched, held against an ambient background, and coverts are pushed out of the way (in order to see the full length of the feather). Sometimes, multiple photos were taken in order to see mites on all nine primaries on each wing or all 12 rectrices (for example, three feathers per photo). A macro‐lens setting on a digital camera was used, and clarity of each photo was checked in the field. No flash was used. A close‐up of the feather mites between feather barbs can be seen in the inset photograph

To quantify mite abundance, we extended the wing and tail of each bird and used a digital camera with a macro‐lens setting to take photos of the ventral side of both wings and both sides of the tail (Figure 2). We reviewed each photo for clarity and compared with the bird in the field to confirm that all individual feather mites across each entire feather were visible before releasing each bird. The process took an average of five min. We uploaded photos to a computer and A.E.M. censused the mites (i.e., counted every individual feather mite) on all 18 primaries (nine on each wing) and all 12 rectrix (tail) feathers.

### 2.6 Reproductive performance

At primary field sites during the breeding season of 2015, we located nests of individuals using behavioral cues (mainly nest building) and monitored them every 1–3 days until fledging or failure. For *P. citrea*, we primarily used digital inspection cameras equipped with flexible, fiber optic cables that can be maneuvered into cavities. We recorded nest content information at each nest including number (and species; both species can be brood parasitized by *Molothrus ater*) of eggs, nestlings, and fledglings. We considered nests active when ≥1 host egg was present. For *S. cerulea*, nest contents were unable to be examined directly until nestlings were visible; therefore, we considered nests active when we observed the female incubating, brooding, or parents provisioning young. Once nestlings neared fledging age (10 to 11 days for both species), we monitored all nests daily to ensure we were able to accurately determine nest fate (failure or fledging). Spotting scopes allowed for monitoring and accurate counting of *S. cerulea* nestlings as they neared fledging age. After presumed fledging occurred, we searched the vicinity around nests for juvenile activity to confirm putative nest fate and to estimate the number of fledglings successfully produced. For nest survival purposes, we considered a nest successful if it produced ≥1 fledgling.

### 2.7 Apparent annual survival

During the 2016 breeding season, we returned to primary field sites to attempt to resight all individuals that were marked the previous year. For males, we visited each territory ≥3 times, and all areas within ~500 m of each territory, and used song‐playback to lure all males into view. In addition, we used song‐playback to lure all males that were heard vocalizing within the greater study areas into view (many from much greater than 500 m from a marked bird's territory). We investigated all previous nest locations to also assist in finding returning *P. citrea* females. Both species have relatively high site fidelity (Boves et al., 2014, 2016; McKim‐Louder, Hoover, Benson, & Schelsky, 2013) and given our level of resighting effort and knowledge of these species, we are confident our methods closely and reasonably approximated annual survival.

### 2.8 Statistical analyses

#### 2.8.1 Hypothesis 1: Mite abundance differs by (a) species, (b) sex, and (c) age

Generalized linear models (GLMs) were built to evaluate how feather mite abundance differs by species (with a focus on nesting ecology; Prediction 1a), sex (Prediction 1b), and age (Prediction 1c) and if any two‐way interactions (*) exist using data collected from both primary and secondary field sites. For this initial modeling attempt, we also included the potentially confounding variables of date of capture, the year of capture (2015 or 2017), and region of capture (north or south) as fixed effects. We removed interactions that were nonsignificant, and then estimated statistics from a model that included the confounding variables listed above. To correct for overdispersion of the data, we constructed GLMs with a quasi‐Poisson error structure and logarithmic link.

If, from this initial modeling attempt, species (or any interaction with species) was an important predictor (Prediction 1a; α = 0.05), we separated species and assessed factors for within‐species differences in mite abundance (Prediction 1b: sex; Prediction 1c: age; and a sex*age interaction) and included the confounding variables of date of capture and region of capture as fixed effects. We did not include year in models with species separated because region and year of capture were perfectly collinear within species (e.g., every *S. cerulea* caught in 2017 was from the southern portion of their breeding range). Again, we removed interactions that were nonsignificant and estimated statistics from a model that included the confounding variables listed.

#### 2.8.2 Hypothesis 2: Relationship between mite abundance and body condition/fitness differs by species and, within species, sex

We used a variety of statistical methods to evaluate the relationships between mite abundance (predictor) and body condition and fitness (responses) for individuals that we followed from 2015 to 2016 (at primary field sites only). We compared inferences between and within species by building separate models for each species and sex (for *P. citrea*). We used this method rather than simply including interactions between mite abundance and species/sex because we were interested in the more subtle differences in the directionality and/or strength of the relationship (between species and between sexes within species).

#### 2.8.3 Prediction 2a: The relationship between mite abundance and body condition will be more parasitic for the cavity‐nesting species and, within this species, females

To estimate body condition, we regressed mass on wing length and then used the resulting residuals as a proxy for body condition (Schulte‐Hostedde, Zinner, Millar, & Hickling, 2005). For each species and both sexes in *P. citrea*, GLMs were constructed and we included the potentially confounding variables of capture date and age in the models as fixed effects in the models. We used a normal distribution and identity link. We also tested for a quadratic relationship between mite abundance and body condition because some evidence suggests that a hormetic, nonlinear relationship may exist (Galván et al., 2008).

#### 2.8.4 Prediction 2b: The relationship between mite abundance and fitness will be more parasitic for the cavity‐nesting species and, within this species, females

For reproduction, we used the Nest Survival module in Program MARK (Dinsmore, White, & Knopf, 2002; White & Burnham, 1999) to evaluate the relationship between mite abundance and daily nest survival for each species and sex. Akaike's information criterion (corrected for small sample size; AIC_c_) was used to compare candidate models to our null models, which for *P. citrea* included nest type (natural or artificial), presence of brood parasitism, age of parent, and geolocator status (if a geolocator had been deployed prior to the completion of the nest) as covariates, and for *S. cerulea* included only the age of the parent. We included the variable of geolocator because for an unrelated study, geolocators (typically <3% of body mass) were attached to 18 *P. citrea* late in the breeding season (after all first broods were complete). *P. citrea* nests that failed due to flooding were excluded from analyses, as this is a stochastic event unrelated to any effect mites may have. Within each sex, for individuals that had >1 nesting attempt during the breeding season, one nest was randomly chosen to include in analyses to maintain independence. A quadratic relationship between mite abundance and nest survival was also examined.

To further assess potential relationships between mite abundance and reproduction, GLMs were built for each species and sex to evaluate the relationship between feather mite abundance and the number of fledglings produced. *P. citrea* can produce two (rarely three) broods. Because we wanted to best capture the entire reproductive history of each individual (rather than selecting a random nest), but we were unable to follow a large enough sample of birds to be confident of their seasonal fecundity, we instead compared the average number of fledglings produced per parent monitored during the season (as opposed to including all nests with individual bird ID as a random variable, for which the models would not converge). The potentially confounding variables of nest type (natural cavity or artificial nest box), age (second year or after second year), presence of brood parasitism, and geolocator status were all included as fixed effects in the models for *P. citrea*, and age alone was included as a fixed effect in the model for *S. cerulea*. A Poisson distribution with logarithmic link was used for this analysis because the data did not follow a normal distribution and could not be normalized. A quadratic relationship between mite abundance and number of fledglings was also examined.

For annual apparent survival, GLMs were built for each species and sex to evaluate the relationship between feather mite abundance and apparent annual survival status (yes or no), and the potentially confounding variables of geolocator status (for *P. citrea*) and age were included as fixed effects in models. A binomial family and logit link were used for this analysis. A quadratic relationship between mite abundance and apparent annual survival was again examined. All statistical analyses, with the exception of nest survival analysis, were performed using the R package “lme4” (Bates, Mächler, Bolker, & Walker, 2014; R Core Team 2016), and all graphics were created with the R package “ggplot2” (Wickham, 2009). All means are reported ± one standard error.

## 3 RESULTS

In 2015, we captured 18 *S. cerulea* (17 males and one female) and 92 *P. citrea* (42 males and 50 females) at primary field sites. In 2017, we captured 11 *S. cerulea* (all males) and nine *P. citrea* (all males) at secondary field sites. Total mite abundance (primaries and rectrices combined) on individual birds ranged from two to 2,254 mites ($\overset{¯}{x}$ = 436 ± 44 mites per individual).

### 3.1 Feather mite identification

Mites from both feather tracts on both host species were morphologically very similar. They are all in the same subfamily (Analgoidea: Proctophyllodidae: Pterodectinae) and the same genus (*Amerodectes*). Genetic data (from the COI gene) suggest that *P. citrea* wing and tail mites are of the same species, but are a different species from *S. cerulea* wing and tail mites, which included two haplotypes of another undescribed species (all are in the process of being described; Matthews et al., in press).

### 3.2 Hypothesis 1: Mite abundance differs by (a) species, (b) sex, and (c) age

**Figure 3:**
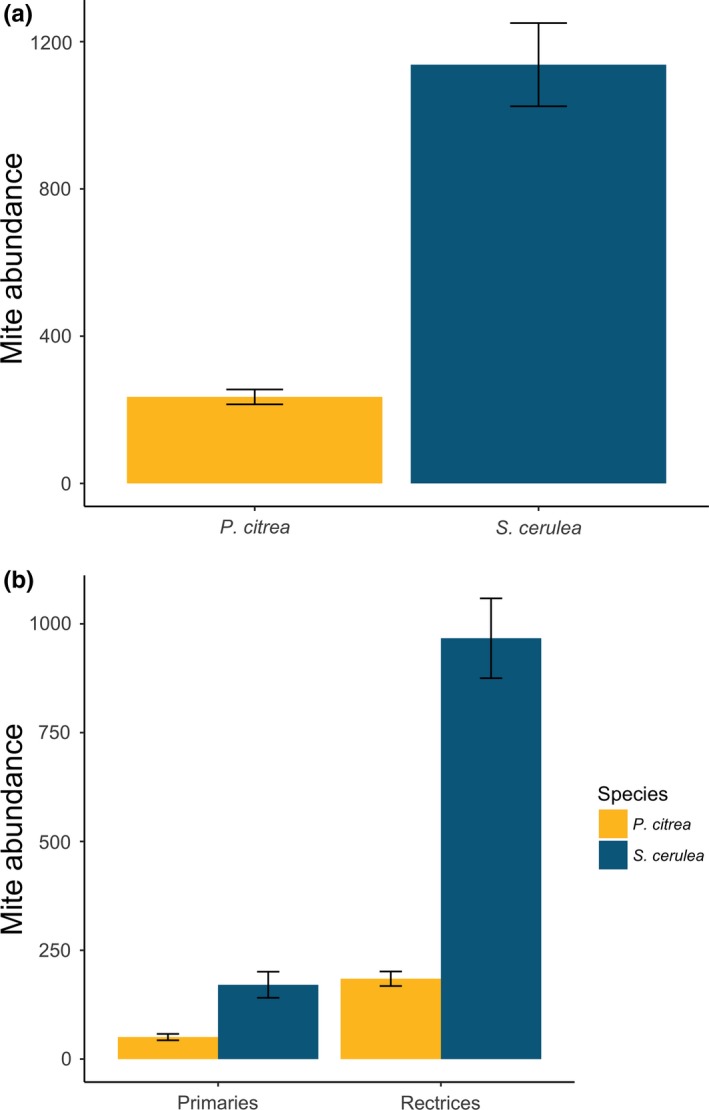
(a) Total average feather mite abundance differed between species: *Setophaga cerulea* (*n* = 29) and *Protonotaria citrea* (*n* = 101; *p* < .001) across both capture years and field sites. Error bars represent ± 1 standard error. (b) Total average feather mite abundance by species and feather tract. *S. cerulea* (*n* = 29) harbored more mites than *P. citrea* (*n* = 101) across both capture years and field sites on both feather tracts, particularly so on the rectrices (*p* < .001), but also on the primaries (*p* < .001). Error bars represent ± 1 standard error

Species was a significant predictor of mite abundance, with *S. cerulea* harboring significantly more mites than *P. citrea* (Prediction 1a; *S. cerulea*: 1,137 ± 113 mites; *P. citrea*: 235 ± 20 mites; *t*_121_ = −7.37, *p* < .001; Figure 3a). While species*age was a significant predictor in this initial modeling attempt (*t*_121_ = −2.55, *p* = .01), the confounding variables of date of capture (*t*_121_ = −0.921, *p* = .36), year of capture (*t*_121_ = 0.52, *p* = .60), and region of capture (*t*_121_ = 1.58, *p* = .12) were all nonsignificant predictors. *S. cerulea* harbored more mites overall, but particularly so on rectrices (*S. cerulea*: 997 ± 91 mites on rectrices; *P. citrea*: 185 ± 17 mites on rectrices; *t*_121_ = −7.42, *p* < .001; Figure 3b). *Setophaga cerulea* also harbored more mites on primaries (*S. cerulea*: 170 ± 30 mites on primaries; *P. citrea*: 50 ± 7 mites on primaries; *t*_122_ = −4.34, *p* < .001; Figure 3b).

**Figure 4:**
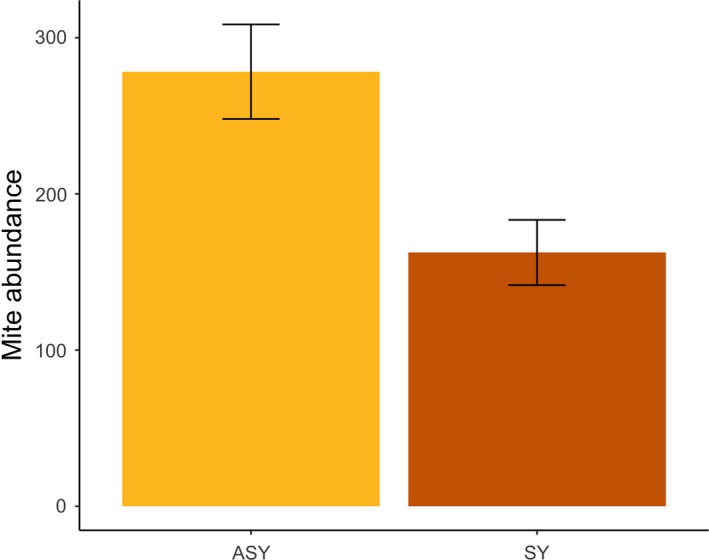
Average feather mite abundance differed between age classes (ASY: after second year; SY: second year) in *Protonotaria citrea* (*n* = 101; *p* = .006) across both capture years and field sites. Error bars represent ± 1 standard error

Because species and species*age were important predictors in the initial modeling attempt, we then separated species and assessed factors for within‐species differences in mite abundance. For *P. citrea*, sex*age was not significant (*t*_94_ = −1.71, *p* = .09), so we removed it from the final model. For *P. citrea,* mite abundance did not differ between sexes (Prediction 1b; males: 248 ± 32 mites; females: 221 ± 24 mites; *t*_95_ = −0.11, *p* = .91), but it did differ between age classes, with older birds harboring more mites than younger birds (Prediction 1c; ASY: 278 ± 30 mites; SY: 162 ± 21 mites; *t*_95_ = −2.81, *p* = .006; Figure 4). The confounding variables of region (*t*_95_ = 0.65, *p* = .52) and date of capture (*t*_95_ = −1.03, *p* = .31) were both not significant. Because only one *S. cerulea* female was captured, we excluded her (as well as sex and sex*age) from analyses. Thus, for *S. cerulea* males, mite abundance did not differ between age classes (Prediction 1c; ASY: 1,102 ± 132 mites; SY: 1,253 ± 189 mites; *t*_25_ = 0.54, *p* = .59). The confounding variables of region (*t*_25_ = 1.31, *p* = .20) and date of capture (*t*_25_ = −0.19, *p* = .85) were both not significant in the model for *S. cerulea* males.

### 3.3 Hypothesis 2: Relationship between mite abundance and body condition/fitness differs by species and, within species, sex

There was no relationship (all *p* > .19) between feather mite abundance (linear or quadratic) and body condition for either species or sex when including age and date of capture as fixed effects (Table 1).

**Table 1:** Summary of results from linear (L) and quadratic (Q) general linear models describing the relationship between feather mite abundance and body condition, offspring fledged, and apparent annual survival by each host species (*Setophaga cerulea* and *Protonotaria citrea* from primary field sites) and sex (male and female). Other confounding, fixed variables that were included in each model are also listed under the respective model. These included date of capture, nest type (natural or artificial), presence of brood parasitism, age of individual, and geolocator status (if a geolocator had been deployed prior to the completion of the nest). Significant results (α = 0.05) are boldface (only seen in confounding effects)

	*Setophaga cerulea* males				*Protonotaria citrea* males				*Protonotaria citrea* females			
	β ± *SE*	Test Statistic	*df*	*p*	β ± *SE*	Test Statistic	*df*	*p*	β ± *SE*	Test Statistic	*df*	*p*
Body condition (L)												
Total mites	<0.001 ± <0.001	*t* = 0.38	11	.71	<0.001 ± <0.001	*t* = 0.44	38	.66	<−0.001 ± <0.001	*t* = −0.74	44	.46
Julian	0.01 ± 0.008	*t* = 2.06	11	.06	<0.001 ± <0.001	*t* = 0.06	38	.95	−0.006 ± 0.007	*t* = −0.83	44	.41
Age	0.23 ± 0.15	*t* = 1.56	11	.15	−0.06 ± <0.19	*t* = −0.30	38	.76	−0.12 ± 0.26	*t* = −0.46	44	.65
Body condition (Q)												
Total mites	<−0.001 ± <0.001	*t* = −0.97	10	.36	0.001 ± <0.001	*t* = 1.34	37	.19	<0.001 ± <0.001	*t* = 0.64	43	.52
Total mites^2^	<0.001 ± <0.001	*t* = 1.11	10	.29	<0.001 ± <0.001	*t* = −1.27	37	.21	<−0.001 ± <0.001	*t* = −0.96	43	.34
Julian	0.01 ± <0.001	*t* = 2.17	10	.06	<0.001 ± 0.004	*t* = 0.09	37	.92	−0.006 ± 0.007	*t* = −0.89	43	.38
Age	0.15 ± 0.16	*t* = 0.97	10	.35	−0.004 ± 0.20	*t* = −0.02	37	.98	−0.15 ± 0.27	*t* = −0.56	43	.58
Offspring fledged (L)												
Total mites	<−0.001 ± 0.001	*Z* = −0.29	3	.77	<0.001 ± <0.001	*Z* = 1.29	29	.19	<−0.001 ± <0.001	*Z* = −1.86	43	.06
Nest type	—	—	—	—	−0.06 ± 0.21	*Z* = −0.33	29	.74	0.06 ± 0.16	*Z* = 0.42	43	.67
Age	−0.99 ± 0.68	*Z* = −1.47	3	.14	0.59 ± 0.23	*Z* = 2.60	29	**.009**	0.15 ± 0.17	*Z* = 0.92	43	.36
Brood parasitism	—	—	—	—	−17.2 ± 1484	*Z* = −0.01	29	.99	−2.15 ± 0.59	*Z* = −3.7	43	**<.001**
Geolocator status	—	—	—	—	0.17 ± 0.32	*Z* = 0.55	29	.58	0.18 ± 0.22	*Z* = 0.83	43	.41
Offspring fledged (Q)												
Total mites	0.009 ± 0.007	*Z* = 1.46	2	.14	<0.001 ± 0.001	*Z* = 0.04	28	.97	<0.001 ± <0.001	*Z* = 0.94	42	.35
Total mites^2^	<−0.001 ± 0.001	*Z* = −1.59	2	.11	<0.001 ± <0.001	*Z* = 0.46	28	.64	<−0.001 ± <0.001	*Z* = −1.60	42	.11
Nest type	—	—	—	—	−0.09 ± 0.22	*Z* = −0.45	28	.65	0.07 ± 0.22	*Z* = 0.47	42	.64
Age	−0.002 ± 0.008	*Z* = −0.31	2	.75	0.57 ± 0.23	*Z* = 2.46	28	**.01**	0.15 ± 0.17	*Z* = 0.92	42	.36
Brood parasitism	—	—	—	—	−17.2 ± 1484	*Z* = −0.01	28	.99	−2.2 ± 0.59	*Z* = −3.67	42	**<.001**
Geolocator status	—	—	—	—	0.19 ± 0.32	*Z* = 0.61	28	.54	0.07 ± 0.16	*Z* = 0.80	42	.42
Survival (L)												
Total mites	−0.003 ± 0.002	*Z* = −1.48	14	.14	0.002 ± 0.002	*Z* = 0.96	38	.34	<0.001 ± 0.002	*Z* = −0.02	45	.98
Geolocator status	—	—	—	—	−0.79 ± 0.98	*Z* = −0.81	38	.42	1.92 ± 0.99	*Z* = 1.94	45	**.05**
Age	−0.82 ± 1.67	*Z* = −0.49	14	.63	1.41 ± 0.94	*Z* = 1.49	38	.14	1.89 ± 0.75	*Z* = 2.53	45	**.01**
Survival (Q)												
Total mites	−0.009 ± 0.007	*Z* = −1.26	13	.21	<0.001 ± 0.006	*Z* = 0.18	37	.85	<0.001 ± 0.006	*Z* = 0.16	44	.87
Total mites^2^	<0.001 ± <0.001	*Z* = 0.92	13	.36	<−0.001 ± <0.001	*Z* = 0.19	37	.84	<−0.001 ± <0.001	*Z* = −0.18	44	.86
Geolocator status	—	—	—	—	−0.79 ± 0.98	*Z* = −0.81	37	.42	1.92 ± 0.99	*Z* = 1.94	44	**.05**
Age	−1.76 ± 2.28	*Z* = −0.77	13	.44	1.37 ± 0.97	*Z* = 0.37	37	.16	1.88 ± 0.75	*Z* = 2.49	44	**.01**

At primary field sites in 2015, we located and monitored 61 nests for *P. citrea*; at 24 nests, we captured both the male and the female; at 26 nests, we only captured the female; and at 11 nests, we only captured the male. We located and monitored five nests for *S. cerulea* males. Mite abundance was unrelated to daily nest survival for both species and both sexes within *P. citrea*; in all cases, the null model was either the best fit model or explained patterns equally as well as the best fit model (Table 2). Mite abundance was also unrelated to the average number of young fledged for both species and sexes (all *p* > .06; Table 1). Finally, mite abundance was unrelated to apparent annual survival of *P. citrea* for both sexes (both *p* > .34), as well as for *S. cerulea* males (*p* = .14; Table 1).

**Table 2:** Candidate models describing the relationship between mite abundance (linear and quadratic) and daily nest survival, by host species (*Setophaga cerulea* and *Protonotaria citrea* from primary field sites) and sex (male and female) For *S. cerulea*, the null model included age of parent. For *P. citrea* males and females, the null model included nest type (natural or artificial), presence of brood parasitism, age of parent, and geolocator status (if a geolocator had been deployed prior to the completion of the nest). The difference between the model with the lowest Akaike information criterion corrected for small sample size (AIC_c_) and each additional model is given (∆AIC_c_). The weight of evidence in favor of a model (*w*_i_) and the number of parameters in the model (*k*) are also given

	*Setophaga cerulea* males				*Protonotaria citrea* males				*Protonotaria citrea* females			
	β ± *SE*	∆AIC_c_	*w*_i_	*k*	β ± *SE*	∆AIC_c_	*w*_i_	*k*	β ± *SE*	∆AIC_c_	*w*_i_	*k*
Null	–	0.06	0.42	2	–	0.40	0.35	5	–	0.00^a^	0.64	5
Null + mite	0.0008 ± 0.002	2.04	0.15	3	0.002 ± 0.002	0.00^b^	0.42	6	0.0002 ± 0.001	1.99	0.24	6
Null + mite + mite^b^	−0.0001 ± 0.00	0.00^c^	0.43	4	0.000004 ± 0.000004	0.12	0.23	7	−0.000006 ± 0.000006	3.24	0.13	7

a AIC_c_ = 129.17.

b AIC_c_ = 93.83.

c AIC_c_ = 20.15.

## 4 DISCUSSION

The nature of the relationship between feather mites and their hosts has recently been debated and previous studies have led to opposing conclusions, while factors that explain this variation remain mostly unstudied. Here, we used two closely related but ecologically distinct Neotropical–Nearctic migratory wood‐warblers to test hypotheses related to factors that may explain variation in (1) feather mite abundance and (2) the relationships between feather mite abundance and host fitness.

Feather mite abundance differed between the two species, supporting our first hypothesis. However, it was in the opposite direction of our Prediction 1a, as *S. cerulea* harbored more feather mites than *P. citrea*. There are a variety of nonexclusive explanations for why *S. cerulea* harbored greater mite abundances, although all will require more study in order to provide strong support. Ecology could have affected mite abundances both directly and indirectly. Both on breeding and wintering grounds, *S. cerulea* live in the overstory canopy of forests, while *P. citrea* occupy the forest understory. These microhabitats differ by many abiotic factors, including temperature and humidity, which could directly affect the ability of mites to survive or reproduce on their hosts (Meléndez et al., 2014; Wiles et al., 2000). It is also possible that because canopy species may be exposed to harsher environmental elements (e.g., rain, wind, and fluctuating ambient temperatures) than understory species, they may need to preen more often to maintain feather condition, thus providing more uropygial oil for mites to consume (Henson, Galusha, Hayward, & Cushing, 2007). Differences associated with the geographic locations of our study areas did not appear to be influential in driving the species differences, as *S. cerulea* from both regions were infested with much greater numbers of mites than *P. citrea* in either region. However, a broad‐scale study involving multiple host species found across latitudinal clines will be useful in determining how much geography (directly or indirectly) influences mite abundances, especially in comparison with other factors such as ecology, phylogeny, and evolutionary history (both within and across host species).

Another possible explanation for the variation in mite abundances between species is behavioral, particularly related to nest materials selected by these species. There is evidence from other cavity‐nesting species (*Sturnus vulgaris* and *Cyanistes caeruleus*) that some green nest materials (mainly angiosperms) that *P. citrea* use in nest lining may be toxic to certain invertebrates and thus reduce ectosymbiont load (Dubiec, Góźdź, & Mazgajski, 2013). *Setophaga cerulea* rarely, if ever, use green materials in their nest building (Buehler et al., 2013; T.J.B., personal observation). This idea could be tested experimentally by adding or removing more toxic (to ectosymbionts) green materials from nests of *P. citrea* and assessing mite abundance among treatments.

In other studies, a strong morphological predictor of feather mite abundance is uropygial gland size, both within and among avian species (Galván et al., 2008; but see Pap, Vágási, Osváth, Muresan, & Barta, 2010). However, it is not a good explanation in this case. We measured the surface area of uropygial glands from a sample of male birds of each species; *S. cerulea* actually had smaller uropygial glands (*P. citrea *= 24.0 ± 0.4 mm^2^; *S. cerulea *= 19.0 ± 0.3 mm^2^) which is expected given that they are smaller overall (body mass of *P. citrea* = 14.11 ± 0.08 g; *S.  cerulea* = 9.71 ± 0.07 g). It is also possible that chemical composition of uropygial gland oil may differ between species, promoting different abundances of feather mites (Haribal et al., 2005). Further investigations of species‐specific anatomical and biochemical traits will be necessary to decipher what proximate mechanisms could influence variation in feather mite abundance.

Related to feather mite abundance within species, our results partially supported one prediction (with respect to age) and refuted the other (with respect to sex). As predicted, older *P. citrea* of both sexes harbored more mites than their younger counterparts. For *P. citrea*, older birds may have simply had a longer amount of time to acquire mites (and for mites to reproduce) than younger birds. These results are consistent with the previous studies of Barn Swallows (Blanco & Frías, 2001; Pap et al., 2005), but inconsistent with results of House Finches (Davis & Cornelius, 2013; Hamstra & Badyaev, 2009), further suggesting that species biology or ecological context are potentially important factors in explaining variation among feather mite studies. We found no difference in feather mite abundance between sexes (of *P. citrea*). However, given our finding that *P. citrea* harbor less mites than *S. cerulea*, it is not unexpected. If canopy, open‐cup nesting species are, in general, more prone to greater mite abundances, future studies should compare mite abundances between sexes of *S. cerulea* and other species of both canopy and understory species.

Although mite abundance varied both between and within species, these patterns do not seem to reflect differential effects of mites on host body condition, reproductive performance, or apparent annual survival, as abundance was unrelated to any of the metrics tested. Overall, these results suggest a commensal relationship between feather mites and these two species, as other studies have also found (Dowling et al., 2001; Galván et al., 2012). This may reflect that these feather mites are simply consuming a minimal amount of uropygial oil, which has little to no impact on the condition or survival of the individual. However, there may be a nonlinear relationship involving a threshold effect of mites (Galván et al., 2008; Haribal et al., 2005), where only individuals with the absolute greatest number of mites are negatively impacted. The possibility of a nonlinear relationship between mite abundance and fitness makes the survival results involving *S. cerulea* of potential continued investigation. Despite a lack of statistical significance, our power to detect a trend was somewhat low as only a small proportion of birds returned to the study area (*n* = 3). Of these three individuals that returned, two of them harbored the two lowest mite abundances of all *S. cerulea*, while six *S. cerulea* with the greatest abundances did not return. In the future, evaluating potential causative effects that feather mites have on hosts would best be explored experimentally by decreasing the number of mites on some individuals (by removal) and comparing reproduction and survival to control groups. It would be difficult to experimentally increase mite abundances on individuals, but this would, hypothetically, be ideal to also include in an experimental design. Another limitation of our study in this regard is that only feather mites were considered; examining (and controlling for) the full symbiont community on hosts (such as nest mites, wing and body lice, and even endoparasites) would help us to better understand how host body condition and fitness can be influenced by multiple symbionts interacting on hosts.

Although not directly related to our hypotheses, differences in mite abundance between feather tracts are, to our knowledge, unique and potentially have implications for future research on feather mite symbioses. Previous studies that have estimated both wing and tail feather mite abundance have not found major differences between the tracts (Pap et al., 2005; Stefan et al., 2015) and Behnke et al. (1999) suggested that tail feather mites are trivial when quantifying feather mite abundances. However, in the present study, we found that mite abundance on rectrices was greater than on primaries for both species (despite possessing more primary feathers) and because the difference between the tracts was even greater for *S. cerulea*, drove much of the variation in mite abundance between species (see Figure 3a,b). This pattern is somewhat surprising as rectrices (in these and most other passerines) are dropped much more readily than primary feathers (T.J.B., personal observation), which if remaining on a rapidly dropped feather, would likely cause mortality of mites. However, greater mite abundance on rectrices in these species could be related to a number of proximate or ultimate factors.

Proximately, rectrices may provide a greater abundance of resources (uropygial oil) for feather mites if birds preferentially preen these feathers, and tail feathers may also experience less turbulence than wing feathers, providing more protection for feather mites, as has been suggested for feather lice (Rózsa, 1993). It is also possible that, ultimately, because feather mite species may differ by feather tract (Fernández‐González, Pérez‐Rodríguez, de la Hera, Proctor, & Pérez‐Tris, 2015) abundances differ due to differential reproductive rates or intraspecific competition (e.g., ideal despotic vs. ideal free distribution). However, this is not likely in our case because, for each of these host species, feather mites from the wing and rectrices were of the same (host‐specific) species in the genus *Amerodectes* (Matthews et al., in press). No matter the proximate or ultimate cause for differential abundances, our data suggest that mite abundances obtained from rectrices can in fact be informative, and because quantification of tail mite abundance does not require any major extension to field methods outlined here, we recommend that rectrices be included in future studies of feather mites on live birds.

In conclusion, we found that *S. cerulea* (a canopy dwelling, open‐cup nesting species) harbored greater abundances of mites than *P. citrea* (an understory dwelling, cavity nester), particularly so on the rectrices. This contradicts our specific prediction, but supports our general hypothesis that feather mite abundance differs between these two ecologically disparate species. Secondly, our data overall support a commensal symbiosis between feather mites and both of these host species. To further improve our understanding of these highly specialized symbiotic systems, future studies should aim to evaluate mite abundance (and the relationship to fitness) on additional host species of varying ecological affinities across their geographic distributions and incorporate experimental tests by removing mites from hosts.

## CONFLICT OF INTEREST

None declared.

## AUTHOR CONTRIBUTIONS

A.E.M. and T.J.B. conceived the initial project ideas and designed methodology. A.E.M., T.J.B., J.L.L., and S.H.S. secured funding for the project. A.E.M., T.J.B., D.W.R., M.C.S., and S.H.S. collected the data. A.E.M. analyzed the data with input from T.J.B.; A.E.M. and T.J.B. led the writing of the manuscript. All authors contributed to editing and revising the manuscript and gave final approval for publication.
